# The speaker behind the voice: therapeutic practice from the perspective of pragmatic theory

**DOI:** 10.3389/fpsyg.2015.00817

**Published:** 2015-06-11

**Authors:** Felicity Deamer, Sam Wilkinson

**Affiliations:** Department of Philosophy, Durham UniversityDurham, UK

**Keywords:** pragmatics, voice-hearing, psychosis, avatar therapy, relating therapy, communication

## Abstract

Many attempts at understanding auditory verbal hallucinations have tried to explain why there is an auditory experience in the absence of an appropriate stimulus. We suggest that many instance of voice-hearing should be approached differently. More specifically, they could be viewed primarily as hallucinated acts of communication, rather than hallucinated sounds. We suggest that this change of perspective is reflected in, and helps to explain, the successes of two recent therapeutic techniques. These two techniques are: Relating Therapy for Voices and Avatar Therapy.

## Introduction

In this paper, we examine the phenomenon of auditory verbal hallucinations (AVHs) from the perspective of pragmatic theory, the branch of linguistics that studies communication^1^. Many attempts at understanding AVHs have tried to explain why there is an auditory experience in the absence of an appropriate stimulus. In other words, they try to explain why certain people have an auditory experience in the absence of the usual conditions for having an auditory experience. Along with many clinicians, we think that voice-hearing should be understood differently. More specifically, we suggest it could be viewed, in many of the most troubling cases, primarily as a hallucinated act of communication, rather than a hallucinated sound. This is in keeping with the idea that human beings are not just perceptual creatures: they are deeply social and communicative. We suggest that this theoretical change of perspective has been foreshadowed by various therapeutic approaches. Furthermore, we suggest that this may provide theoretical insights into the apparent successes of recent therapeutic techniques. We examine two such techniques: *Relating Therapy for Voices* (Hayward et al., 2009) and *Avatar Therapy* (Leff et al., 2014).

We proceed as follows. We start by explaining the shift in focus that we endorse, namely, from audition to communication. Then we show how pragmatic theory, which examines how human beings extract meanings from utterances, might be applied to AVHs, conceived in the way we propose. Finally, we present the two therapeutic techniques mentioned above and give an explanation of why they might be effective from the perspective of pragmatic theory.

## From Audition to Communication

Standard theoretical attempts to explain AVHs tend to try to explain why there is an auditory experience in the absence of an appropriate stimulus. This clearly builds on a particular characterization of the phenomenon to be explained. Thus, for example, Garrett and Silva (2003, p. 445) state that “the subjective quality of sensation is a near-universal feature of auditory hallucinations.” Wu (2012, p. 90) states that, “in every instance of AVH, the relevant internal state is a type of auditory state.” Even the well-known PSYRATS scale, which in many respects is in keeping with our approach, describes AVHs as:

An auditory perception that has the compelling sense of reality of a true perception but occurs without external stimulation of the relevant sensory organ.

(Haddock et al., 1999)

Our suggestion is that, for many of the most distressing AVHs, we should not focus on sounds and on auditory experiences, but rather on communication. In other words, these hallucinations are most fruitfully viewed as *communication hallucinations* that happen to be auditory, rather than *auditory hallucinations* that happen to be communicative.

We think that this position is motivated by the fact that an auditory-based approach mischaracterised several instances of the phenomenon. Firstly, some voice-hearers describe an experience of “soundless voices” (reports of which go at least as far back as Bleuler, 1950). For example, one participant in a recent study reports:

It’s hard to describe how I could ‘hear’ a voice that was not auditory; but the words used and the emotions they contained were completely clear, distinct and unmistakeable, maybe even more so than if I had heard them aurally.

(Woods et al., 2015, p. 326)

Secondly, “voices” are also “heard” by congenitally deaf people with psychosis. Jo Atkinson (2006) has done very important work correcting the “audio-centrism” of mainstream perspectives. She has shown that deaf voice-hearers experience vague visual imagery like being addressed in sign-language, or of disembodied lips. But they do not have auditory experiences at all.

Of course, somebody might object that in congenitally deaf “voice-hearers,” the visual imagery is the basis of the “voice” hallucination, in a way that is analogous to how, on an audition-centered view, the auditory experience forms the basis of voice-hearing experiences in voice-hearers who aren’t deaf. This strikes us as implausible, in part because it fails to account for reported cases, which we have just mentioned, among the hearing population, of soundless voices, or experiences of “receiving a communication without any sensory component” (Frith, 1992, p. 73). Furthermore, it fails to make obvious use of what “voice-hearing” experiences in deaf and hearing populations have *in common*. We suggest that what they have in common is that they involve the experience of being communicated with. Once you do this, you can account for the differences relatively easily, since the communication is bound to be experienced in the way that communication tends to be experienced by that individual (viz., signing for the deaf). This also accounts for why there is a much higher prevalence of hallucinations reported in the auditory modality (within the hearing population) among subjects with psychosis, namely, because they tend to communicate auditorily, with speech (clearly there are hallucinations and sensory disturbances in other modalities, too, be we are explaining the *relative prevalence* of auditory hallucinations).

Another phenomenon, found in 64% of inpatients suffering from a psychotic disorder (Minas et al., 1992) which may also benefit from something like our approach, is delusions of reference. As Startup and Startup (2005) note, there seem to be at least two varieties of delusions of reference^2^. One variety concerns the mistaken sense that others are communicating by subtle non-verbal means (e.g., gestures, stances, arrangements of objects), while the other variety concerns the false belief that others are stealthily spying. It is the former kind that may both benefit from, and support, a communication-centered approach. The factors that may then account for the differences between communicative AVHs and communicative delusions of reference would be an interesting direction for research, but goes beyond the scope of this brief paper.

It is important, at this point, that we be clear as to which subset of the heterogeneous group of phenomena that get called AVHs we think are the most suitable to being viewed as communication hallucinations (that sometimes happen to be auditory). First of all, there are auditory hallucinations that aren’t strictly AVHs, because they are not verbal (e.g., groans, clicks, bangs, music). However, among the clearly verbal cases, there is the issue of whether the subject is perceived as the specific addressee of the speech act. Cases where this is most clear are when the voice is either commanding, critical or abusive (these features are reported in Nayani and David, 1996 with a prevalence of 84, 77, and 70%, respectively). Cases where this is less clear, are cases where the voice is giving a neutral, running commentary (e.g., “John is raising his right arm and standing up to pick up the paper” etc.). Although these cases can be recognized as self-generated, they can be distressing because they are loud and interfere with the voice hearer’s life^3^. These kinds of voices are not the kinds of voices that we think are best captured by our approach and it is worth noting that the kind of distress caused indirectly through the voice interfering with the subject’s life is rather different from the kind of distress directly caused by the voice-hearer being abused, criticized and commanded by a malevolent agent. The prevalence of running commentary hallucinations is relatively high, but not as high as, for example, command hallucinations (55% vs. 67% in McCarthy-Jones et al., 2012). It is the latter, along with abusive and critical hallucinations, that are best captured by our communication-centered approach.

## Pragmatic Theory and the Extraction of Meaning from Speech

The usual conditions of someone speaking, and one’s understanding them, do not only involve the sounds that they make, but also a grasp of what they intend to communicate. The study of communication is the territory of “pragmatic theory,” the branch of linguistics that studies “utterances” (namely, linguistic communicative acts) rather than free-floating, de-contextualized, “sentences.”

It is important to recognize that a voice-hearing experience is the experience of a spoken utterance, not a free-floating sentence. On hearing an utterance, a hearer will automatically interpret the meaning of that utterance. It is this interpretation process, which we think is important for explaining why there so often is a speaker (e.g., a person, a demon, or a god) behind the voice. In order to interpret the meaning of an utterance, a hearer must attribute the intentions behind its use at that moment, and intentions are never free-floating: they are always the intentions of an agent, of something with a mind (Wilson and Sperber, 2004).

A voice-hearer might hear the utterance “He is a loser.” Without knowing whom the speaker intends to refer to with the pronoun ‘he,’ the hearer can’t know what is meant by that utterance. And yet it seems that voice-hearers generally know who is being referred to in their voices, and what the voices mean. As Waters and Jardri (2015, p. 534) put it: “The clarity of hallucinated speech is indeed often low or fuzzy although the message is always clear to the patient.”. This suggests that the voice-hearer takes there to be a speaker behind the voice, with an intention to communicate, and they interpret what it is that speaker is saying; who they are referring to, and why.

Likewise, a voice-hearer might hear a voice say “Nice one!” The meaning of this will depend entirely on the intention perceived. It could either be something positive, an expression of congratulation. Alternatively, it could be sneering and sarcastic. Somebody might object that this is something that the voice-hearer will tell from the way that the utterance is said, and ultimately from what it sounds like. But we suggest that ‘the way it is said’ or ‘what it sounds like’ is determined by the emotion of the speaker, e.g., elation, in the positive case, or, e.g., shame, in the negative case. This means that the fact that it is a person (a communicator) behind the voice, and that they are nasty, explains why the utterance sounds the way it does, and thus, why it is interpreted the way it is, rather than the other way around.

Within this approach, where voice-hearing experiences are primarily viewed as communicative rather than auditory, it becomes less surprising that the voice is taken to come from a speaker (communicative agent), since this is a necessary dimension of all communication. Going beyond suggestions from Bell (2013) and Wilkinson and Bell (forthcoming), it is relevant to say not only that representation of agency in general, and specific agents in particular, is a key aspect of many AVHs: but also that the representation of agents *as communicative*, and as *affording communicative interactions* is also crucial.

## A Perspective on Therapeutic Interventions

Although clear and detailed theoretical formulations of this approach have been lacking, it is implicit (and at times explicit) in a number of recent therapeutic frameworks and techniques (see Thomas et al., 2014 for a review). Here we examine two promising such techniques: *Relating Therapy* and *Avatar Therapy*.

### Relating Therapy

#### What is It?

Relating Therapy was developed by Hayward et al. (2009) with the aim of improving the relationship between voice hearers and their voices by encouraging them to interact closely with their voices and to relate to them in a more assertive manner.

A Relating Therapeutic intervention consists of multiple sessions, which make up three separate phases. The aim of phase 1 is to introduce the voice hearer to the notion of Relating Therapy, and to consider the implications of relating to and interacting with their voices. The objective of phase 2 is then to explore the themes within the voice hearer’s history with regard to how they have related socially with other people as well as with their voices. Having completed phase 1 and 2, the voice hearer is then ready to enter into phase 3 by exploring and developing assertive approaches to relating to and socially interacting with their voices and other people. Together with the therapist, the voice hearer participates in role-plays to explore the intentions and motives of their voices and other people, and to practice a more assertive approach to social interaction.

#### Why Does It Work?

Relating Therapy was developed based on the intuition that, just as our perceptions of, reactions to and relationships with our colleagues, our family members and our friends are effected by how we relate to those individuals, a voice hearer’s perception of, reaction to and ultimately their relationship with their voices might be affected by how they relate to those voices. For example, a voice hearer might assume a passive and submissive role in relation to their voice, thus allowing the voice to assume a dominant, bullying role. Hayward et al. (2009) conceived of the therapy as a tool for normalizing the voice-hearing experience by locating it in the realm of typical social interactions. They recognize the disparity between hearers’ reported emotional responses to their voices (Garrett and Silva, 2003) and their passive relationship with them, and they intend the therapy to be a route out of this relationship and into one of a more reciprocal nature. Indeed, findings from an early feasibility study suggest that Relating Therapy reduces the distress caused by voices and triggers a change in how the hearers relates to their voices (Hayward et al., 2009).

Relating Therapy in its conception embraces the agentive aspect of the voice-hearing experience, and encourages voice hearers to view their voices as coming from intentional agents whose behavior is dependent on how the voice hearer relates to them. Indeed, when AVHs are viewed as intentional communicative acts, the speaker behind those communicative acts is more specifically a communicative agent who seeks to influence and bring about emotions and behaviors in the hearer via the production of (often) distressing utterances. As such, the voice hearer has the capacity to become an interlocutor in the experience and talk back to the speaker behind their voice, potentially bringing about change in the content of their verbal hallucinations, and ultimately empowering the voice hearer by exposing them to the malleability of their voice-hearing experiences and their emotional responses to them. After all, a communicative exchange is an iterative process in which what one interlocutor says builds on what has already been said and influences what will then be said by the other interlocutor.

### Avatar Therapy

#### What is It?

Avatar Therapy consists in encouraging the voice hearers to construct a visual “avatar,” using a computer program. The voice hearer subsequently interacts with the avatar. In a pilot study, after up to seven 30 min sessions of Avatar Therapy, almost all of the patients reported an improvement in both the frequency and the severity of their voices. Three of the 16 patients stopped hearing voices completely after up to 16 years of experiencing AVHs.

#### Why Does It Work?

The intention behind the development of Avatar Therapy was to develop a tool to facilitate a dialog between voice hearers and their voices. Leff et al. (2014) propose that an avatar provides something for the voice hearer to stand up to, and, ultimately, something that they can control. As is pointed out in Leff et al. (2014), many clinicians are skeptical of this therapeutic approach, because it is seen as an extreme case of colluding with the patient’s pathology, providing a ‘real’ physical manifestation of that which is hallucinated. Like Leff et al. (2014), we suggest that this line of thought is misguided. Leff et al. (2014), defend their approach by emphasizing that:

Since the externalized voice is part of the patient’s inner world, discounting it or refusing to acknowledge the patient’s experience of this split-off part as real negates the possibility of the patient reintegrating it into their psychic structure.

(Leff et al., 2014, p. 6)

We want to unpack this point a little further by emphasizing that when AVHs are viewed primarily as hallucinations of intentional communicative acts, the communicator behind the voice can be as much part of the experience as the sound of the voice, or as what the voice is saying. In fact, we suggest that the latter two aspects of the experience are likely to be dependent on the voice hearer’s perception of the speaker behind their voice. It is implicit in the thinking behind Avatar Therapy that the speaker behind the voice is critical to the voice hearer’s perception of and reaction to their voice, but Leff et al. (2014, p. 1) see “the entity” behind the voice as “invisible” in AVH. We suggest that quite the opposite to providing a physical manifestation of that which was ‘invisible’ before, Avatar Therapy affords the voice hearer the opportunity to have some control over the characteristics of the speaker behind their voice in a controlled and deliberate manner, where, before, the speaker behind the voice, though an ever present aspect of the AVH experience, had free reign to present in any way^4^.

The positive outcomes of Avatar Therapy are not surprising if voice-hearing experiences are seen as primarily communication hallucinations. The distressing aspect of voice-hearing is often the intention behind the voice, i.e., to threaten, harass, or abuse. Indeed, pragmatic theory teaches us that the mere words heard without the underlying (perceived) intentions are not simply *weaker* threats (or harassments, or abuses): they fail to be threats at all. It makes sense that if the voice-hearer is able to create a benevolent avatar for their voices, then the attitude and content conveyed by the voices will invariably be less malicious and less distressing.

## Conclusion

In keeping with recent therapies that seem effective, an important subset of AVHs should be viewed as hallucinations of intentional acts of communication, rather than primarily as hallucinated sounds. This manages to capture what we take to be important in many AVHs that are reported as auditory, as well as those experiences reported as soundless voices, and “voice-hearing” experiences in the congenitally deaf. Theorists who want to explain voice-hearing in terms of, e.g., spontaneous activation in auditory cortex (Cho and Wu, 2013), would either deny the existence of these phenomena, or say that they are not “real instances” of AVH. This latter option, we think, is revisionist and undesireable. We would rather encourage the grouping together of communication AVHs, some of which may be auditory, while others might not be, rather than a grouping along the axis of auditory phenomenology. Of course, this then makes the term “AVH” a bit of a misnomer. But superficial terminological considerations should not be what drives us when we group together clinically relevant phenomena. We could easily call them “verbal hallucinations” or “voice hallucinations,” or even stick to the pre-theoretical term “voice-hearing experiences.”

From our communication-centered perspective, the interpretations hearers derive from these experiences, and the distress they cause, can be better understood. Moreover, such an approach may allow pragmatic theory to provide insights into how a voice-hearer might be able to manipulate the content and delivery of their voices by engineering the identity and nature of the speaker behind the voice.

## Conflict of Interest Statement

The authors declare that the research was conducted in the absence of any commercial or financial relationships that could be construed as a potential conflict of interest.

## Footnotes

1 For the record: FD is a linguist and SW is a philosopher.

2 We would like to thank one of the referees for pointing us in the direction of these communicative delusions of reference.

3 We would like to thank one of the referees for pointing this out.

4 Indeed a voice hearer recently told us: “My voices are always here, looking at you, even when they are silent.”
